# Establishing an intraoperative, mobile CBCT-based workflow for gynecologic brachytherapy: primary experience and benefit assessment

**DOI:** 10.3389/fonc.2025.1562670

**Published:** 2025-04-16

**Authors:** Andre Karius, Vratislav Strnad, Christoph Bert, Rainer Fietkau, Ricarda Merten, Claudia Schweizer

**Affiliations:** ^1^ Department of Radiation Oncology, Universitätsklinikum Erlangen, Friedrich-Alexander-Universität Erlangen-Nürnberg (FAU), Erlangen, Germany; ^2^ Comprehensive Cancer Center Erlangen-EMN (CCC ER-EMN), Erlangen, Germany

**Keywords:** interventional radiotherapy, intraoperative imaging, image-guidance, adaptive brachytherapy, mobile CT

## Abstract

**Background and purpose:**

In the brachytherapy of cervical cancer, creating a suitable implant based on ultrasound guidance may be impacted by imaging limitations. To validate the implant if ultrasound is not sufficient, we implemented a new workflow utilizing additional intraoperative cone-beam computed tomography (CBCT). The aims of this work were to describe the newly established workflow, reflect associated (dis)advantages, and assess geometric and dosimetric benefits compared to the previous solely ultrasound-guided workflow.

**Materials and methods:**

We report the establishment of our new workflow utilizing mobile CBCT during interventions and corresponding experiences for 26 consecutive patients. Image quality was assessed by considering the applicator visualization and contrast–noise ratio (CNR) between tissues. Implant changes based on CBCT scans were analyzed with respect to the enhanced insertion depths (EIDs) of needles and their tip distances to target volume borders. Dosimetric effects were evaluated by calculating common dose–volume parameters for target volume and organs at risk (OARs) and comparing them in both a previous patient cohort and scenarios simulating sole ultrasound guidance. Implant uncertainties between intra- and postoperative imaging were analyzed using a corresponding registration as well.

**Results:**

Implementing intraoperative CBCT was associated with clinical challenges but increased safety feeling during interventions and resulted in geometric as well as dosimetric benefits. Needles could be shifted deeper into the pelvis by an EID of 14 ± 11 mm based on CBCT, associated with corresponding significant dose improvements for target volume and OARs with a mean tradeoff increase of up to 4.8 Gy. With a reasonable CNR between tissues up to 8.5 ± 3.6 and clear detectability of applicators, image quality was sufficient to fulfill intraoperative intentions. Furthermore, the CBCT scans were suitable for treatment planning purposes from a geometric uncertainty perspective.

**Conclusion:**

The implementation of intraoperative CBCT can substantially improve the quality and safety of image-guided gynecologic brachytherapy.

## 1 Introduction

Image-guided brachytherapy is an important cornerstone in the treatment of several gynecologic malignancies such as cervical cancer (1–3). Regarding cervical cancer, brachytherapy is typically delivered as a boost following external beam radiotherapy (EBRT) for the application of high doses to the tumor while sparing surrounding organs at risk (OARs) as best as possible (1, 4). Prospective trials already revealed the very good local control and low extent of toxicity achievable using this technique (5–7).

**Figure 1:**
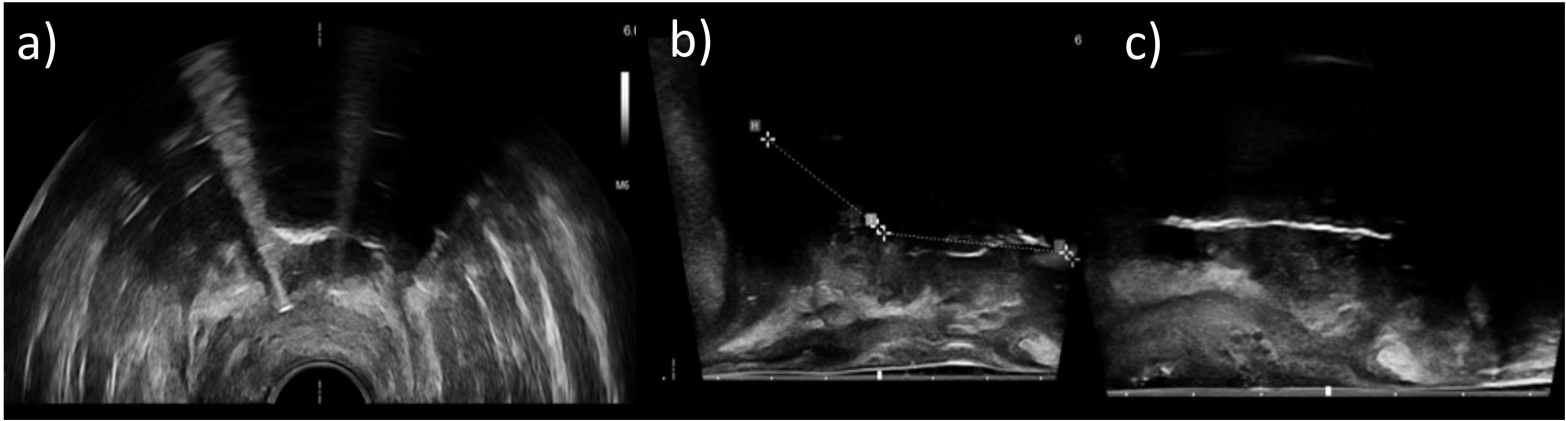
Axial **(a)** and sagittal **(b, c)** transrectal ultrasound images of three different patients acquired during an interventional procedure. It was observed that some image regions only appeared shadowed and provided no information about anatomy or applicator courses due to lack of image contrast. Sufficient imaging with ultrasound alone was not possible in these cases.

To obtain sufficient clinical outcomes, a high-quality image-guided applicator insertion individually adapted to the underlying patient is essential. Only if applicators such as intrauterine probes or interstitial needles are inserted with high accuracy into the target volume will optimized treatment planning and dose delivery become feasible (2, 8, 9). In this respect, most interventional procedures are nowadays guided by transrectal or abdominal ultrasound, as described in current guidelines (10–12). However, although ultrasound provides a good soft-tissue contrast in general, in some cases, corresponding imaging for the purpose of implantation guidance and visualization of interstitially inserted needles is strongly impeded or rendered impossible at all (11, 13–15). This holds especially for implantations deep in the pelvis (16), anatomically difficult circumstances (e.g., seroma occurrence), and laterally extended as well as bulky tumors (11, 13, 14) (**Figure 1**). In these cases, image quality can be deteriorated by the insertion depth or anatomy itself, placed tamponades, or the “shielding” of needles by the ultrasound reflections of adjacent needles/applicators. The aim of achieving a highly accurate, patient-adapted implantation may thus become impracticable.

To overcome this issue, three-dimensional (3D) cross-sectional imaging such as magnetic resonance imaging (MRI) or computed tomography (CT) can be applied in the intraoperative setting (17, 18) but is, to our knowledge, still limited to a few centers worldwide likely for financial and logistical reasons. The mentioned modalities enable a validation of the correct applicator placement *in situ* in 3D and subsequently improve the implant geometry if required. Nevertheless, they are also associated with disadvantages described in literature (17–19), e.g., additional effort for medical staff, the requirement for varying the patient position for fitting into the gantry, or the potential need for repetitive image acquisitions to optimize the implant, which can result in prolonged anesthesia time. The expense associated with corresponding intraoperative workflows therefore has to be evaluated in terms of a cost–benefit assessment with regard to the achievable benefits for patients.

In this context, we recently implemented a new standard workflow for the brachytherapy of cervical cancer utilizing both intraoperative ultrasound and intraoperative mobile cone-beam CT (CBCT). The aims of the present work were to describe our newly established workflow and clinical procedures, reflect our single-center experience, and in this way provide a recommendation for the implementation of similar workflows in other institutions. Furthermore, we conducted an assessment of achievable implant-geometric and dosimetric improvements compared to the previous, solely ultrasound-based workflow. Finally, we evaluated the extent of geometric implant uncertainties associated with our current procedure.

## 2 Materials and methods

### 2.1 Previous workflow without intraoperative CBCT

Prior to the introduction of mobile CBCT, our intraoperative workflow for the brachytherapy of cervical cancer was based solely on ultrasound guidance, as described in the following.

At the beginning of the interventional workflow, the patient was positioned on a non-radiopaque surgical table, and general anesthesia was inducted. After the legs were in the lithotomy position, transvaginal and rectal ultrasounds were conducted as well as palpatory examinations to confirm the extent of the high-risk clinical target volume (HR-CTV) as determined in the brachytherapy pre-planning (performed 1–3 days prior to the intervention based on clinical examinations and cross-sectional imaging). The surgical site was disinfected and covered with sterile drapes, and a bladder catheter was inserted.

In the next steps, the cervix was visualized by means of specula and dilatated by means of Hegar probes under ultrasound guidance, and the length and flexion of both the cervical channel and uterine cavity were measured using a corresponding measurement probe. Based on the findings from all of the examinations, the choice of types, numbers, and dimensions, as well as insertion depths of the applicators, was made, and their respective implantation was performed. In our department, the majority of patients received a Fletcher applicator comprising an intrauterine probe and associated ovoids, which was, in case of laterally extended diseases, accompanied by the interstitial implantation of plastic needles through the ovoid guidance holes using metal mandrins. In case of an additional extent into the vaginal mucosa, an intrauterine cylinder probe and titanium needles were usually applied, which were fixed via a Martinez Universal Perineal Interstitial Template (MUPIT) or Syed template to the patient’s perineum, instead of a Fletcher applicator. Afterward, the applicators were fixed in position by placing corresponding tamponades. Transrectal ultrasound examinations aimed to validate the applicator positioning *in situ*, based on which their locations were corrected if applicable in an iterative manner. This completed the interventional procedure.

After anesthesia was discharged, a corresponding planning-imaging procedure of the patient with applicators *in situ* at distant devices requiring patient transfer was conducted. Images via either CT or MRI were acquired depending on clinical availability. The CT examinations comprised 120-kV tube voltage, automatic exposure modulation, and 0.4 × 0.4 × 2 mm^3^ voxel size. The pMRIs comprised a T2-SPACE sequence with 1 × 1 × 1 mm^3^ voxel size, with MRI-visible markers being placed into the intrauterine probe and ovoids but not into needles. Based on the planning images, the applicator courses *in situ* were reconstructed, and HR-CTV and OARs were contoured following GEC-ESTRO guidelines (11, 20) within the treatment planning system Oncentra Brachy (Elekta, Best, Netherlands). In the subsequent treatment planning, the dwell positions and times of the ^192^Ir source of a microSelectron afterloader (Elekta, Netherlands; step-size 2.5 mm) were manually defined and optimized. The aim of treatment planning was to achieve the best possible HR-CTV coverage with the prescribed dose while being restricted by dose constraints of the rectum (75 Gy EQD2 (dose isoeffective to a fractionation of 2Gy single dose) in combination with the previous EBRT) and bladder (85 Gy EQD2). The details regarding applicator reconstruction, dose–volume histogram analysis, and dose prescription can be found in the International Commission on Radiation Units and Measurements (ICRU) report 89 (21) and the current GEC-ESTRO guidelines (11, 20, 22, 23). The treatment was conducted in the pulsed dose rate (PDR) regime with hourly irradiations of 0.5–0.65 Gy up to a total dose of 40–45 Gy, depending on clinical requirements.

### 2.2 Establishing a new workflow: intention of intraoperative CBCT

An accurate implantation of applicators is important to enable optimized treatment planning and dose delivery. In particular, it has to be noted that due to the applicator design, the first possible dwell position is located several millimeters (approximately 6 mm for intrauterine probes and 5 and 9 mm for plastic and metal needles, respectively) away from the physical applicator tip. A sufficient insertion depth for placing dwell positions as close as possible to the cranial HR-CTV border to ensure a reasonable dose coverage without risking perforations into vessels or intestines located very often directly behind it is therefore crucial. However, based on clinical experience and as mentioned in the introduction, a corresponding adequate validation of applicator positions is not always feasible using ultrasound alone and may be subject to increased uncertainties (11, 13–15, 24) (**Figure 1**). To enhance the evaluation of the applicator arrangement *in situ*, we integrated additional CBCT into the final step of our intraoperative workflow. We chose CBCT since it enables fast 3D imaging of both plastic and metal applicators, is logistically easier and faster to implement than MRI (no requirements for creating an MR-safe surgical environment), and enables image acquisitions with non-moving patient tables, advantageous in intraoperative settings.

For validating the applicator positions, our aim was to perform the CBCT scans directly in the surgical theatre and to have the images immediately available to the physician for further decisions. In all three main planes (axial, sagittal, and coronal) as well as multiplanar reconstructions, examining the centered location of the probe within the uterus (to enable symmetrical irradiations) as well as the sufficient probe depth with their tip being placed at the fundus uteri and (in case of a Fletcher being used) the ovoids being in direct contact with the portio vaginalis uteri was considered important. In the same way, evaluating a suitable depth of interstitial needles with their tips being placed at least at the cranial HR-CTV border (or 5/9 mm across this for plastic/metal needles, see above, in case of no potential risk of OAR injuries) was deemed essential. To facilitate dose planning based on clinical experience, a merging of needle tips on the scans should be avoided, and an equidistant needle spacing was strived for. The final verification and, if required, iterative improvement of applicator positioning in relation to the anatomy prior to ending the intervention was rated important to adapt the implant to the patient-specific requirements.

### 2.3 Mobile CBCT device

For implementing CBCT, we utilized the X-ray system ImagingRing (medPhoton, Salzburg, Austria). This device has already been characterized in previous studies (25–27) and showed particularly high CT number accuracy as well as good high-contrast visualization (25). Recent investigations have also reported improvements regarding geometric stability (27) and described applications for treatment quality assurance (QA) (16). However, the establishment of a corresponding intraoperative brachytherapy workflow as well as associated clinical experiences and benefits have not been outlined and assessed in the literature so far.

The ImagingRing is a mobile device that can perform longitudinal, lateral, and rotational motorized movements and is controlled based on a tablet PC. Its dimensions amount to 182 × 87 × 190 cm^3^, enabling a compact operation in the surgical theatre. Source and a 43.2 × 43.2 cm^2^ large flat-panel detector, which have been characterized previously (25), rotate independently along the gantry of 121-cm clearance, allowing for non-isocentric imaging and imaging with laterally enlarged field of view (26) (FOV). The FOV size can be adjusted to the anatomy of interest based on planar topograms recorded in the anterior–posterior and lateral directions. Dynamic jaws ensure the corresponding X-ray beam shaping during the scanning procedure.

Based on our CBCT experience, the ImagingRing was operated with the following parameters: 120-kV tube voltage, patient-specific tube-current modulation, 12-Hz frame rate, 0.6-mm focal spot, 0.2- or 0.5-mm prefiltering, 300-µm binned detector pixel size, 360° scan mode, 54–66-s acquisition time, 0.6 × 0.6 × 0.6 mm^3^ reconstructed voxel size, and the Shepp–Logan kernel. The aims of the scanning procedure were to address the clinical issues described in Section 2.2 and, thus, ensure a high implant quality based on the acquired images.

### 2.4 Workflow assessment

The establishment of our new workflow, which is reported in the results section as an outcome of our previous procedures (Section 2.1) and the desired objectives (Section 2.2), started in 2024. Since then, 26 cervical cancer patients have been treated and received a sole Fletcher applicator, a Fletcher with additional interstitial needles, and a cylindrical applicator with titanium needles in 8, 16, and 2 cases, respectively. We considered all of these consecutive patients (patient characteristics are provided in **Table 1**) treated in our institution starting from the establishment of our new workflow for the present study, without any further selection criteria. Apart from technical and logistical aspects, especially subjective experiences play a decisive role in rating the quality of a workflow. Therefore, both of these areas were evaluated.

**Table 1:** Patient and treatment characteristics of the patients receiving the CBCT-based workflow.

Parameter	Median (range)
Age (years)	54 (33–83)
Body mass index (BMI) (kg/m^2^)	25 (16–36)
HR-CTV (cm^3^)	21 (7–60)
	Number of patients
FIGO stage	
- FIGO I	2
- FIGO II	10
- FIGO III	13
- FIGO IV	1
Applicators used	
- Sole Fletcher applicator	8
- Fletcher applicator + plastic needles	16
- Cylindrical applicator + titanium needles	2

CBCT, cone-beam computed tomography.

In the first step, the time requirement of each new workflow step was determined and put into perspective in relation to the total intervention time between inducing and discharging anesthesia. Furthermore, the number of required CBCT scans and the associated dose exposure were documented for each patient. For the latter, the weighted cone-beam dose index CBDI_w_ (28) calculated for an IEC 60601-2-44 acrylic 32-cm body dosimetry phantom (29) and provided by the ImagingRing’s control software was considered.

In addition, the subjective experience of our interdisciplinary brachytherapy team comprising senior physicians, physicists, and surgical nurses was collected in several discussions. Based on this, the perceived time and space requirement of using the ImagingRing, the changed atmosphere in the surgical theatre, the safety feeling regarding sufficient implantation accuracy, and the visualization of acquired scans including the delimitability of tissue structures and the applicator reconstructability, as well as the additional effort associated with the new workflow, were described. The reporting and reflection on our collective experiences served as the basis for recommendations regarding the establishment of respective workflows and raising awareness of corresponding (dis)advantages.

### 2.5 Soft-tissue contrast and applicator visualization

**Figure 2:**
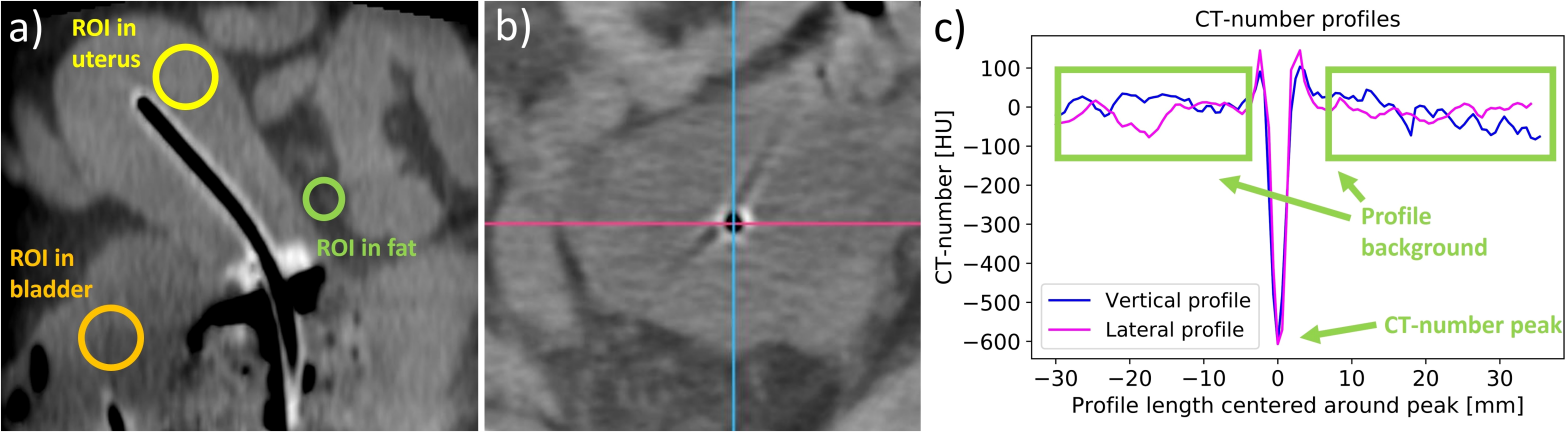
The placement of regions of interest (ROIs) in the uterus, the bladder, and the surrounding fat tissue **(a)**, which were used to determine the contrast–noise ratio. Furthermore, lateral and vertical CT number profiles were measured in the regions of the applicator tips (shown in panel **(b)** as an example of an intrauterine probe) to evaluate the visibility of the applicators. The two directions are exemplified by a lateral pink line and a vertical blue line. The CT number of the peak at the applicator position and the mean value of the CT numbers of the profile background were then calculated from the created profiles **(c)**, and their difference was determined.


To support the subjective image quality evaluations by quantitative measures following Karius et al. (16), we additionally determined the image contrast of the final acquired CBCT scan of each patient. In this regard, we placed a circular region of interest (ROI) each within the uterus in proximity to the intrauterine probe, the bladder, and the uterus-surrounding visceral fat as exemplarily illustrated in **Figure 2**. Based on CT number average #CT  and standard deviation σ of the corresponding ROIs (referred to as x and y in the equation below), the contrast–noise ratio CNR between the bladder and uterus as well as the uterus and fat was determined (16) by **Equation 1**:

(1) $$CNR_{x-y}=\frac{\left|\#CT_{x}-\#CT_{y}\right|}{\sqrt{\frac{1}{2}\cdot \left(\sigma_{x}{\;}^{2}+\sigma_{y}{\;}^{2}\right)}}.$$

Moreover, lateral and vertical CT number line profiles were drawn across the location of each probe and needle tip in the axial slices, as shown in **Figure 2**. The tips were chosen since their visualization was the main reason for implementing intraoperative CBCT due to the limited access of ultrasound imaging to these regions. The average and standard deviation of the profile background (excluding adjacent applicators and bones) were calculated and compared to the CT number of the profile peak observed at the respective applicator location. The statistical significance of the peak amplitude exceeding the background was tested using a one-sided one-sample t-test at a significance level of 5%. This served as a measure for the clarity of applicator visualizations using CBCT.

### 2.6 Assessment of geometric benefits

**Figure 3:**
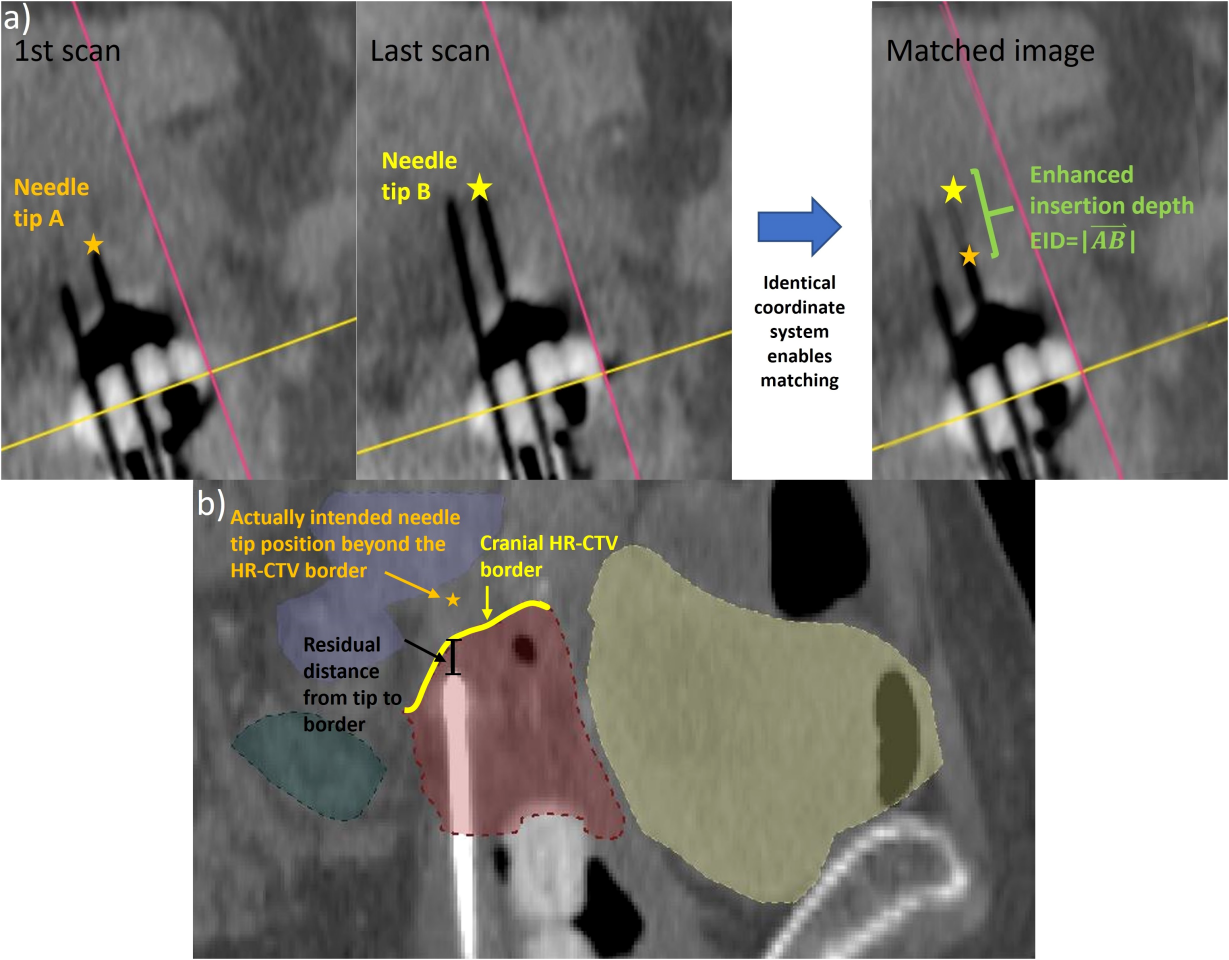
To determine the enhanced insertion depth (EID) as a measure for the applicator shift performed based on the intraoperative cone-beam computed tomography (CBCT) scans **(a)**, the tip of each varied applicator was identified on the first and last intraoperative CBCT scans. Since the images were acquired in the same coordinate system, the EID could be determined directly from a corresponding match. Furthermore, the residual distance of each applicator that was not implanted to the cranial high-risk clinical target volume (HR-CTV) border **(b)** to this border along the implantation direction was calculated as a measure of implant inaccuracies.


To analyze the geometric improvements regarding implantation accuracy achievable with intraoperative CBCT, the number of applicator position changes conducted considering the CBCT images were documented for each patient at first. In cases where CBCT-based adjustments had to be made, the distances that the individual needles were shifted deeper into the pelvis to obtain the desired needle tip position (see above) were measured by comparing the situation on the last acquired scan to the initial scan (the latter referred to the situation resulting from sole ultrasound guidance). For this purpose, all applicators were reconstructed on the corresponding images to obtain their 3D courses *in situ*. Note that the CBCT series of each specific patient was acquired in the identical CBCT coordinate system, and, hence, it became feasible to directly overlay the individual scans. Based on this image fusion, the 3D vector $\vec{AB}$ between the tip A reconstructed on the last CBCT and tip B reconstructed on the first CBCT was determined for each applicator (**Figure 3**). The enhanced insertion depth (EID) resulting from using intraoperative CBCT was then calculated according to **Equation 2** to

(2) $$EID=\{\begin{array}{c} +\;\left|\vec{AB}\right|,\;if\;needle\;has\;been\;shifted\;deeper\\ -\;\left|\vec{AB}\right|,\;\;\;\;\;if\;needle\;has\;been\;retracted \end{array}$$

Furthermore, the suitable placement of needle tips *in situ* was assessed considering the treatment planning imaging (section 2.1). As described in Section 2.2, needles should have been implanted with their tips being at least located at the cranial HR-CTV border or even shifted across it. For all needles not fulfilling this demand, the distance between the respective tip and the cranial HR-CTV border along the implantation direction was measured. The same measurement was also conducted for the last 25 patients treated with the previous, solely ultrasound-based intraoperative workflow to identify improvements in this respect. The significance of differences was assessed by means of Welch’s t-test at a significance level of 5%. The conducted analysis served to estimate the geometric implant inaccuracies occurring in both the previous and newly established workflows.

### 2.7 Assessment of dosimetric benefits

To assess the dosimetric effects of the workflow change, the treatment plans delivered to the patient (Section 2.1) were evaluated. In particular, the dose parameters D_90,CTV_ (dose the most exposed 90% of the HR-CTV receive), V_100,CTV_ (HR-CTV receiving at least 100% of the prescribed dose), and D_2ccm_ of the bladder and rectum were considered. Furthermore, to analyze the tradeoff between exposure to OARs and target volume, the ratio D_2ccm_/D_90,CTV_ was calculated for both the bladder and rectum and indicated the dose that these structures received per unit D_90_ applied. To enable a dose comparison across individual fractionation schemes, all volume and dose metrics were reported in percentage of the HR-CTV and prescribed dose, respectively.

Potential improvements resulting from the implementation of intraoperative CBCT were assessed in a retrospective cohort-based analysis first. For this purpose, the aforementioned parameters were determined from the treatment plans of the 26 consecutive patients who received the newly established workflow as well as of the last 25 patients who received the previous, solely ultrasound-based implantation procedure. The results were compared by means of a Welch’s t-test at a significance level of 5%.

Moreover, a prospective dosimetric analysis was performed by virtually shifting each needle reconstructed on the planning imaging for exactly the distance EID determined in Section 2.6 reverse to the direction it was shifted considering the CBCT scans during surgery. All patients that required an implant adjustment were considered in this evaluation. This served to simulate the implant arrangement resulting from sole intraoperative ultrasound guidance prior to performing the CBCT-based adjustments. The virtual shift was conducted by changing the individual offset in the treatment planning system, i.e., the distance between the reconstructed needle tip and the first possible dwell position (note that this value is normally set to −5 mm for plastic or −9 mm for metal needles due to the needle design, as explained above; for instance, if a plastic needle should be virtually retracted by 10 mm in the caudal direction, a corresponding offset of −15 mm would have been inserted). Blinded to the actual clinical treatment plan delivered to the patient, an additional treatment plan was calculated by a physicist for the simulated scenario and cross-checked by a physician. The plans for the simulated scenarios were compared to the clinical plans considering the parameters mentioned above. Significance was tested using a paired two-sample t-test at a significance level of 5%. This procedure served to estimate the dosimetric improvements achievable for individual patients by implementing CBCT into our intraoperative workflow.

### 2.8 Identification of implant uncertainties

The reliability of intraoperative CBCT scans in terms of an accurate representation of the applicator geometry *in situ* in relation to the anatomy is considered important. In this respect, the implant visualized on the CBCT scans should match the implant on the planning imaging as closely as possible to ensure that any intraoperative adjustments could be transferred into an optimized treatment planning process. However, uncertainties such as image distortions (24, 25, 30) or geometric variations caused by patient position change or transfer (31, 32) could contradict this aim.

To investigate this issue, all applicators reconstructed on the final intraoperative CBCT scan (Section 2.6) and the planning imaging were resampled (33) with a sampling distance of 0.5 mm between the manually set reconstruction points. Based on a variation of the Iterative Closest Point Algorithm (34), the resampled intrauterine probe of the CBCT scans was rigidly registered to the respective probe course visualized on the planning imaging. This procedure was conducted assuming a fixed applicator position within the uterus due to the placed tamponades. To assess the registration quality, the mean absolute error (MAE) between all N resampled and registered reconstruction points RP_CBCT_ and RP_pim_ of the CBCT scan and the planning imaging, respectively, was then calculated according to **Equation 3**:


(3) $$MAE=\;\frac{\sum_{n=1}^{N}\left|RP_{CBCT,n}-RP_{pim,n}\right|}{N}.$$

Based on this registration, the courses of all other applicators (i.e., ovoids and needles) were compared between both image data sets by calculating the MAE as described above as well. Occurring differences in applicator courses served as indirect measures for both actual applicator arrangement changes *in situ* (e.g., due to patient re-positioning) and geometric uncertainties associated with the intraoperative CBCT scans with respect to the postoperative planning images.

## 3 Results

### 3.1 Establishment of the new workflow

**Figure 4:**
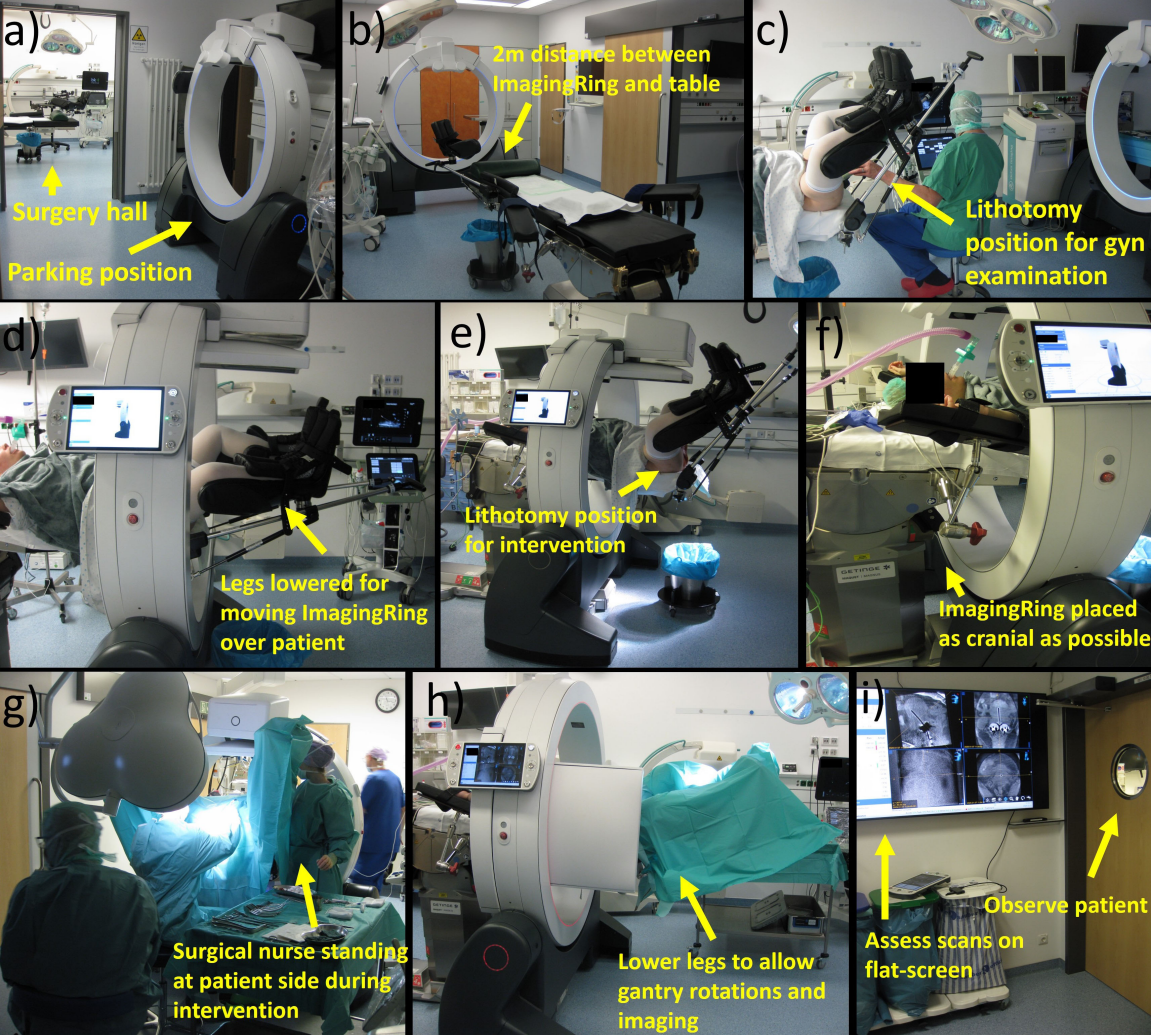
Depicted is a representation of the individual workflow steps. Prior to surgery, the ImagingRing is moved from its parking position to intervention room **(a)** at a distance of 2 m from the foot-end of the table **(b)**. After performing a gynecologic examination in lithotomy position **(c)**, the legs have to be lowered to move the ImagingRing over the patient **(d)**. Afterward, the lithotomy position is set again to perform the intervention **(e)**. The device has to be moved as cranially as possible **(f)** to allow surgical nurses to stand at the patient’s side during the intervention **(g)**. At the end of the procedure, the legs have to be lowered again for imaging **(h)**. The scans are assessed on a large flat screen in an adjacent room **(i)** under simultaneous patient observation. Afterward, corresponding implant adjustments associated with lifting and lowering legs and repetitive imaging may be performed before moving the ImagingRing back to its parking position and ending surgery.

Establishing a new intraoperative standard workflow for the brachytherapy of cervical cancer in our institution formed a major result of the present work. Its final realization is described as follows and illustrated in **Figure 4**.

At first, implementing CBCT into our routine procedure started even prior to accompanying the patient into the surgical hall by moving the ImagingRing from its parking position in an adjacent room to approximately 2 m in front of the foot end of the surgical table. This position was selected to save time by avoiding ImagingRing movements into the room during surgery and to provide enough space and time for the medical staff for subsequent anesthesia induction, bringing the patient into lithotomy position, and performing the initial gynecologic examinations as in the previous workflow. After these examinations and prior to the disinfection step (Section 2.1), the patient’s legs were lowered again to move the CBCT device over the patient in the cranial direction as far as possible (i.e., until it almost hit the table base). This enabled working on both sides of the patient during the interventional procedure, e.g., for disinfection, covering the surgical site, or the surgical nurses holding the specula. In this respect, special focus was placed on the patient’s arms, which had to be positioned with bent elbows and the forearms running along the cranio-caudal direction to avoid collision with the ImagingRing. Afterward, the patient was brought back into the lithotomy position, and the interventional procedure was continued according to the previous ultrasound-guided routine (Section 2.1).

After completing the final transrectal ultrasound examination at the end of the previous workflow (Section 2.1), an additional ImagingRing scan was acquired to visualize the applicators *in situ*. For this, the patient’s legs had to be lowered again a bit to enable collision-free gantry rotations, and the table height (if required) as well as the ImagingRing’s longitudinal position needed to be adjusted to place the pelvis exactly into the imaging isocenter. After these adjustments, a dry-run gantry rotation of 360° was performed to ensure the collision-free movements of the source and detector around the patient. Thereafter, the medical staff entered an adjacent radiation-protected room (with a view into the surgical theatre to observe the patient) equipped with a 65-inch flat screen, and topograms as well as a subsequent CBCT scan were acquired. While viewing and analyzing these images is by default only feasible at the ImagingRing’s operation tablet PC, an HDMI cable connection was utilized to transfer the view to the flat screen and enable a corresponding large-scale image visualization. Based on this, the applicator arrangement *in situ* in relation to anatomy was assessed in all three main planes and multiplanar reconstructions to investigate the issues mentioned in Section 2.2.

In case implant changes were required, the medical staff again entered the surgical hall, performed the desired changes, and acquired a further CBCT scan in the same ways as described above. This procedure was conducted in an iterative manner until a sufficient applicator arrangement was created. Finally, the patient’s legs were brought back into a flat-lying position, the ImagingRing was moved to its parking position in the adjacent room, anesthesia was discharged, and the patient was repositioned into the hospital bed. Afterward, the procedure continued as in our previous workflow with external planning imaging performed for subsequent treatment planning.

### 3.2 Workflow assessment

The additional CBCT-related steps of the newly established workflow required altogether an average (± standard deviation) duration of 18.6 ± 8.9 min (range, 8.9–43.6 min). This referred to 28% ± 12% (range, 10%–58%) of the total intervention time, which amounted to 67 ± 20 min (range, 38–111 min) considering all patients treated so far. The time needed to move the ImagingRing from its parking position into the surgery hall (mean, 4.3 ± 0.4 min) prior to surgery was thereby excluded. For a comprehensive summary of the time requirements of all implemented steps, please refer to **Table 2**. It has to be noted that the times for performing a CBCT scan and adjusting the implant are provided in this table for a single event, i.e., in case of repetitive CBCT scans acquired for a patient, the corresponding time sum had to be considered. In this respect, four, three, and two imaging procedures were conducted for one (4%), three (12%), and nine (35%) patients, respectively. In 13 (50%) cases, the control CBCT imaging served to confirm a sufficient implant quality created by sole ultrasound guidance, and no implant adjustments were required (these cases were neglected in providing a mean value for this step in **Table 2**). However, in eight of the 13 cases, only a Fletcher applicator was implanted, and, hence, 62% of all patients receiving interstitial needles obtained at least one implant adjustment based on intraoperative CBCT. The scans were associated with a mean CBDI_w_ of 9.2 ± 1.3 mGy (range, 6.7–11.8 mGy), resulting in an average dose exposure of 15.4 ± 7.1 mGy (range, 7.1–28.4 mGy) in the patients considering repetitive imaging.

**Table 2:** Listed are the individual workflow steps that were implemented into our intraoperative workflow.

CBCT-related workflow step	Time requirement
Move ImagingRing from parking position to surgery hall	4.3 ± 0.4 min (range, 3.5–5.4 min)
Move ImagingRing over the patient into position desired for performing the intervention	0.8 ± 0.2 min (range, 0.4–1.2 min)
Set correct imaging position and perform dry-run rotation	3.3 ± 0.5 min (range, 2.0–4.2 min)
Acquire topograms and single CBCT scan and perform image assessment (including time for leaving and entering the surgical theatre)	5.6 ± 1.0 min (range, 3.5–7.5 min)
Adjust implant based on single CBCT scan	3.0 ± 0.6 min (range, 1.9–4.0 min)
Move ImagingRing from surgery hall to its parking position at end of surgery	2.3 ± 0.6 min (range, 0.5–3.0 min)

The time requirement shows the respective mean and standard deviation of the time an individual step took as well as the corresponding range (minimum to maximum) in brackets.

CBCT, cone-beam computed tomography.

The time requests for the additional workflow steps were perceived as a challenge in our clinical routine, especially in case of several surgical procedures scheduled a day. In particular, the repeated leaving and entering the operating theatre as well as changing the leg positions were considered to disrupt the otherwise smooth procedure. Furthermore, the loud noise of the ImagingRing’s cooling fans was experienced as a disruptive factor during the entire surgery, and the device led to a slight restriction of the personal working space of the surgical nurses standing at the patient’s sides, e.g., holding the specula, despite the device’s mobility and flexibility in positioning. However, the physicians were not negatively affected by the performance of the intervention in any kind. In the opinion of our entire team, the described challenges were outweighed by the benefits achieved with the intraoperative CBCT imaging. In each case and in particular in cases with complex anatomy, the additional scans strongly increased the safety with respect to a correct applicator positioning *in situ*. For the implantation of interstitial needles, partly several CBCT-based adjustments (see above) of the correct needle depths—that were considered unfeasible using ultrasound alone—were performed. CBCT imaging was, therefore, especially in case of laterally extended diseases, considered beneficial for creating a suitable patient-adapted implant with high confidence. In this respect, the quality of the workflow would have felt substantially downgraded without visualizing the scans on the large flat screen, as viewing them on the ImagingRing’s small tablet PC was deemed cumbersome.

**Figure 5:**
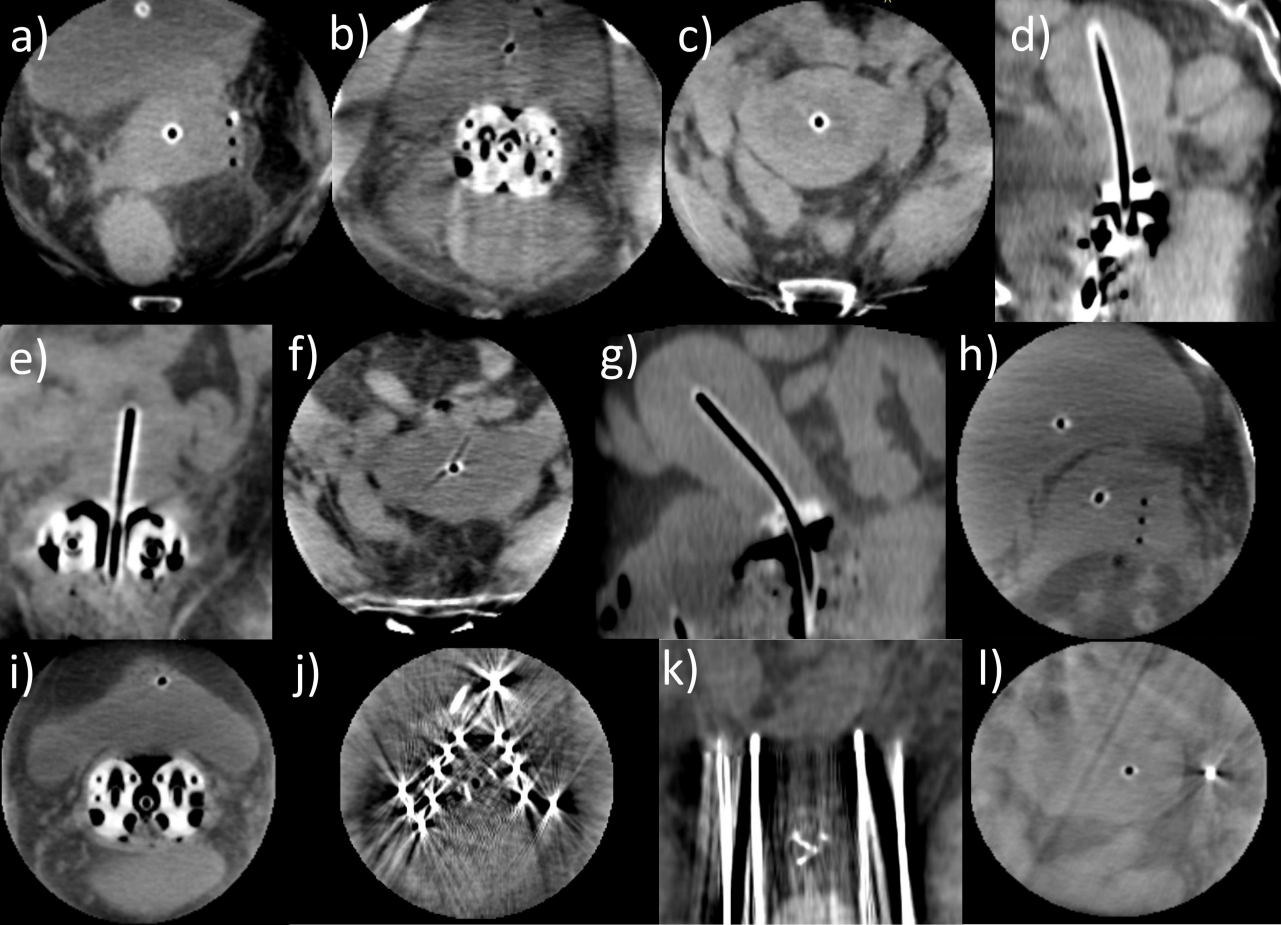
Several cone-beam computed tomography (CBCT) scans acquired with the ImagingRing as an example of the good image quality achieved that was sufficient for our intraoperative purposes **(a–i)**. In these cases, tissue structures could be clearly distinguished, and the applicator courses *in situ* in relation to the anatomy were well identifiable. However, in case of metal needles being applied, the tissue around the needles was completely blurred **(j)** due to metal artifacts, but the needle depths could still be evaluated to a limited extent **(k)**. Nevertheless, using the ImagingRing in combination with metal needles was deemed insufficient. Furthermore, on a few scans, image artifacts resulting from scatter and motion of the bowel **(l)** impacted image quality.

In terms of image quality, a clear visualization of applicator courses and especially needle tips *in situ* sufficient for our intraoperative purposes was achieved (**Figure 5**). Soft-tissue structures and surrounding organs such as the uterus, bladder, and intestine could be reasonably distinguished for 20 patients. No dependency on the patients’ body mass index (**Table 1**) was observed with respect to image quality. However, a strict delineation was impacted by reduced tissue contrast or scatter/motion artifacts originating from intestinal gas bubbles as well as peristaltic movements in six patients who received a Fletcher applicator with or without additional plastic needles. Metal needles could still be identified exactly using an adequate CT number windowing but also caused strong artifacts, blurring the surrounding tissue and making the delineation of structures within the corresponding image regions almost unfeasible. Using the ImagingRing in combination with metal needles was therefore deemed inadequate. Nevertheless, intraoperative CBCT imaging provided in each case added value to pure ultrasound-guided implantation with respect to the assessment of applicator courses *in situ* in relation to the anatomy.

### 3.3 Soft-tissue contrast and applicator visualization

The preceding descriptions were supported by the quantitative analysis of tissue contrast and applicator visualization. Considering the intraoperative CBCT scans, we obtained a mean contrast–noise ratio (CNR) of 5.1 ± 3.2 (95% confidence interval [CI] for mean value: [3.9; 6.3]) between the bladder and uterus as well as 8.5 ± 3.6 (95% CI: [7.1; 9.9]) between the uterus and visceral fat. These measurements confirmed the subjective impression of a reasonable differentiability of tissue structures for most patients (see above), despite representing only exemplary calculations. The image contrast was thus by a factor of approximately 5 and 8 higher than the corresponding image noise level, allowing for a sound assessment of the applicator placement in relation to anatomy. Exceptions were given by the scans affected by artifacts as reported above, resulting in observations of a minimum CNR of 1.0 and 2.6 between the bladder and uterus as well as the uterus and fat, respectively. This highlighted the importance of reducing corresponding artifacts to ensure good image quality.

Considering the CT number profiles drawn across the applicators confirmed the good high-contrast visualization on the scans as well. The profile peaks could be clearly and significantly (p < 0.001) distinguished from the corresponding image background with average CT number differences of 419 ± 184 Hounsfield units (HU) (range, 20–1,067 HU; 95% CI: [387 HU; 451 HU]). These results showed the benefits of CBCT for visualizing especially the applicator tip locations (where the profiles were drawn according to Section 2.5) exceeding the possibilities of sole ultrasound guidance in some cases as reported above. Summarizing the results of Sections 3.2 and 3.3 and considering the mentioned exceptions, the utilized CBCT scans were in general associated with a reasonable image quality sufficient for their intraoperative purpose.

### 3.4 Geometric benefits

**Figure 6:**
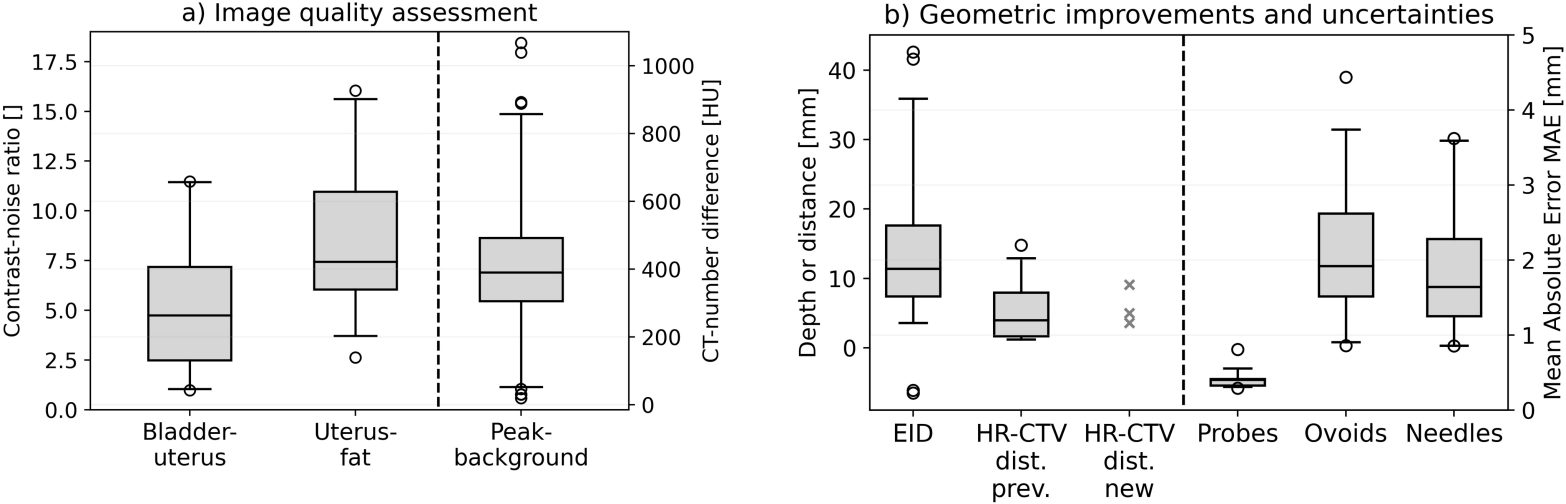
The results obtained in analyzing the contrast–noise ratio between bladder and uterus as well as between uterus and visceral fat. The Hounsfield unit differences between the CT number peaks at the applicator locations and the image background are illustrated as well **(a)**. Furthermore, the enhanced insertion depth (EID) achieved with the cone-beam computed tomography (CBCT)-based workflow and the distance between the needle tips featuring an insufficient insertion depth to the cranial high-risk clinical target volume (HR-CTV) border for both the previous workflow (HR-CTV dist. prev.) and the implemented CBCT-based workflow (HR-CTV dist. new) are provided **(b)**. Since only three needles had to be considered for the latter, the concrete measurement results instead of a boxplot are shown for this case. Finally, the mean absolute error (MAE) obtained for intrauterine probes, ovoids, and needles as uncertainty measures are shown. In the boxplots, the horizontal line indicates the median, the boxes the interquartile range, the whiskers the 95th percentile of the results, and the circles the outliers.

The implementation of intraoperative CBCT resulted in substantial geometric benefits regarding an improved applicator placement *in situ*, particularly considering the interstitial insertion of needles. With respect to all considered treatments, a CBCT-based adjustment of the intrauterine probe due to a non-centered location within the uterus or a suboptimal implantation depth was conducted in only one patient, whereas 42 of all 64 implanted needles (66%) required adjustments along the insertion direction. Forty needles had to be shifted deeper into the pelvis, and two needles were retracted in the caudal direction. The mean EID was 14 ± 11 mm (range, −6 to 43 mm; 95% CI: [11 mm; 18 mm]; **Figure 6**), whereby 60%, 33%, and 21% of the affected needle depths varied by >10, >15, and >20 mm, respectively. The feasibility of improving needle depths based on intraoperative CBCT was considered clinically relevant to access also deeper and laterally extended regions as desired with the needles. However, note that while in case of an implant arrangement being changed always a second CBCT scan was acquired for validation, the corresponding corrections of 13 (20% of all implanted needles) and four (6%) needles required a third and fourth scan, respectively, due to tissue variations.

The improvements in implantation depth became also apparent when comparing the patients receiving intraoperative CBCT to the previous cohort. In this regard, the new workflow reduced the number of implanted needles not reaching the cranial HR-CTV border from 18% (12 of 68 implanted needles) with a residual distance to this margin of 5.5 ± 4.7 mm (range, 1.2–14.8 mm; 95% CI: [2.9 mm; 8.1 mm]; **Figure 6**) to only 5% (three of 64 needles, with distances of 3.6, 5.1, and 9.1 mm). Three, two, and none of the affected needles showed still deviations >2.5, >5, and >10 mm, respectively, compared to 12%, 7%, and 3%, respectively, implanted in the previous workflow. For the needles not implanted deep enough, the distance to the cranial HR-CTV border differed not statistically significant (p = 0.87) between the new and previous workflow, but this analysis was impacted by the low number (i.e., statistical sample size) of only three needles affected after the workflow change. However, as mentioned above, the number of affected needles was substantially reduced, highlighting the clinical relevance and importance of considering the intraoperative CBCT scans. Thus, intraoperative CBCT resulted in substantial geometric benefits regarding a suitable applicator placement *in situ*. No dependency on the patients’ body mass index was observed for any evaluated geometric parameter.

### 3.5 Dosimetric benefits

**Figure 7:**
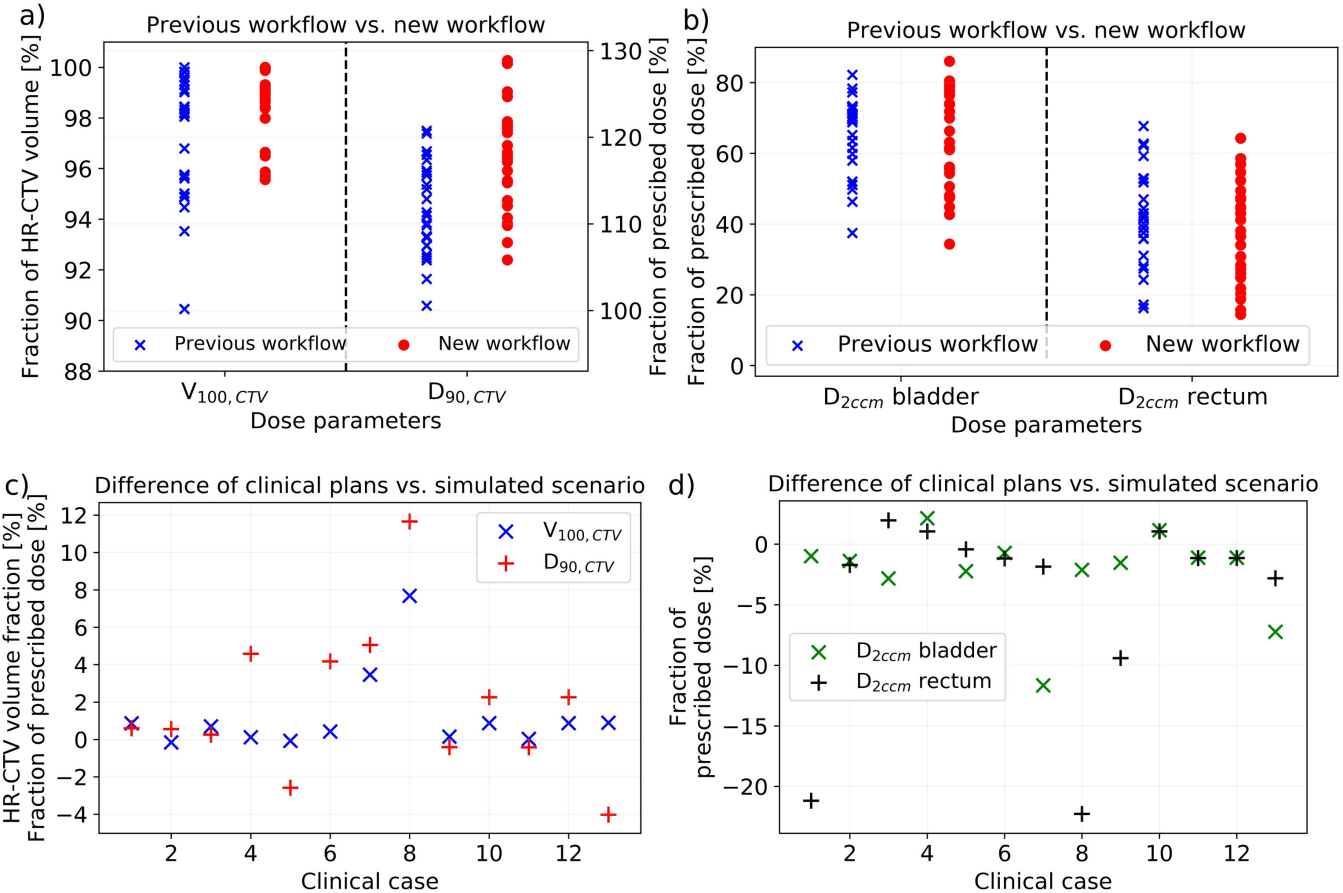
The dosimetric effects of implementing cone-beam computed tomography (CBCT) into our new workflow obtained considering the treatment plans of our patients. In the cohort-based analysis, there was a significant improvement in V_100,CTV_ and D_90,CTV_ **(a)**, whereas only a slight impact on D_2ccm_ rectum **(b)** was observed. The volume results are provided in fraction of the high-risk clinical target volume (HR-CTV) and the dose results in fraction of the prescribed dose. In comparing the clinical treatment plans to the plans created on the scenario of virtually shifted needles simulating the situation after sole ultrasound guidance, only small differences in V_100,CTV_ (again provided in fraction of the HR-CTV) and D_90,CTV_ (in fraction of the prescribed dose) were obtained **(c)**. However, both bladder and rectum D_2ccm_ decreased in most cases when considering intraoperative CBCT **(d)**.

In the cohort-based comparison of dosimetric parameters, a trend to an improved coverage V_100,CTV_ and D_90,CTV_ of the HR-CTV for the patients receiving the new workflow was observed (**Figure 7**). In this respect, V_100,CTV_ changed statistically significantly from 97.2% ± 2.5% (95% CI: [96.2%; 98.2%]) achieved with the previous procedure to 98.7% ± 1.4% (95% CI: [98.1%; 99.3%]) (p = 0.02) and D_90,CTV_ from 111% ± 5% (95% CI: [109%; 113%]) to 117% ± 6% (95% CI: [115%; 119%]) (p = 0.001) of the prescribed dose. The parameter ranges improved from 90.4%–99.6% to 95.7%–100.0% for V_100,CTV_ and from 100%–120% to 106%–129% for D_90,CTV_. Based on the patient-specific adaption of the applicator courses *in situ* using intraoperative CBCT, it thus became feasible to apply higher treatment doses to the target volume. While the cohort-based analysis showed no dosimetric effects on the bladder {D_2ccm_ of 65% ± 11% (95% CI: [61%; 69%]) of the prescribed dose vs. 64% ± 14% (95% CI: [59%; 69%]) previously}, the rectum D_2ccm_ decreased slightly from 42% ± 14% (range, 16%–68%; 95% CI: [37%; 48%]) to 37% ± 14% (range, 14%–64%; 95% CI: [32%; 43%]). Considering a typical brachytherapy prescription dose of 45 Gy, the presented findings referred to a mean D_90,CTV_ increase of 2.5 Gy and simultaneous rectum D_2ccm_ decrease of 2.3 Gy, thus expanding the tradeoff between OAR and target volume exposure by 4.8 Gy. This was considered clinically relevant, especially for patients featuring suboptimal anatomy or reaching corresponding dose constraints. Note that, due to the strong D_90,CTV_ improvements, the normalized OAR exposure D_2ccm_/D_90,CTV_ was decreased for both the bladder (0.58 ± 0.12 (95% CI: [0.54; 0.63]) to 0.55 ± 0.13 (95% CI: [0.50; 0.60]); p = 0.21) and rectum (0.38 ± 0.14 (95% CI: [0.33; 0.44]) to 0.32 ± 0.13 (95% CI: [0.27; 0.37]); p = 0.09). Regarding all evaluated dosimetric parameters, we observed no effects of body mass index differences between patients on the results.

Comparing the clinical treatment plans to the plans created for virtually shifted needles (simulating the scenario of sole ultrasound guidance), dosimetric improvements using intraoperative CBCT were achieved as well (**Figure 7**). All 13 patients requiring an implant adjustment were considered in this evaluation. In this respect, an increase in coverage V_100,CTV_ from 96.7% ± 3.0% (range, 90.3%–99.8%; 95% CI: [95.1%; 98.5%]) for the simulated scenario to 98.0% ± 1.5% (range, 95.6%–100.0%; 95% CI: [97.2%; 98.9%]) for the clinical treatment plans was obtained. D_90,CTV_ increased from 113% ± 7% (range, 100%–121%; 95% CI: [109%; 117%]) to 115 ± 5% (range, 108%–125%; 95% CI: [112%; 118%]). These changes were just not significant (p ≥ 0.08), highlighting that a sufficient dose coverage of the target volume by an enhanced expanding of dwell times of individual dwell positions of the afterloader source is even for a suboptimal implant feasible in principle. However, for individual patients, these changes were partly substantial and beneficial (**Figure 7**). Moreover, the observations were associated with substantial drawbacks for OAR exposure. The bladder D_2ccm_ decreased significantly (p = 0.048) from 73% ± 15% (95% CI: [65%; 82%]) of the prescribed dose for the simulated cases to 71% ± 13% (95% CI: [63%; 78%]) for the clinical plans, and the rectum D_2ccm_ revealed corresponding variations from 48% ± 14% (95% CI: [40%; 56%]) to 43% ± 14% (95% CI: [35%; 51%]) (p = 0.03). For a typical prescription dose of 45 Gy, utilizing intraoperative CBCT thus resulted in D_90,CTV_ increases of 0.9 Gy as well as simultaneous bladder and rectum D_2ccm_ reductions of 1.1 and 2.1 Gy, respectively, which was again considered clinically relevant for individual patients. The normalized OAR exposure D_2ccm_/D_90,CTV_ was decreased for both the bladder {0.65 ± 0.15 (95% CI: [0.57; 0.75]) to 0.62 ± 0.13 (95% CI: [0.54; 0.69]); p = 0.07} and rectum {0.43 ± 0.14 (95% CI: [0.35; 0.51] to 0.37 ± 0.13 (95% CI: [0.31; 0.45]); p = 0.06}. Considering both the cohort-based and individual patient analyses, using intraoperative CBCT resulted in dosimetric improvements compared to the solely ultrasound-guided workflow.

### 3.6 Implant uncertainties

To assess the implant uncertainties associated with considering intraoperative CBCT, the applicator arrangements as visible on the final CBCT images and the planning imaging were evaluated. The registration of the intrauterine probes yielded a mean MAE of only 0.41 ± 0.12 mm (range 0.29–0.81 mm; 95% CI: [0.37 mm; 0.46 mm]; **Figure 6**), referring to a high accuracy of their rigid matching. Based on this, comparing the reconstructed courses of all other implanted applicators such as ovoids and particularly needles enabled the assessment of the relative deviations of these applicators on the planning imaging to their arrangement observed on the final CBCT images. In this respect, we determined a mean MAE of 2.0 ± 0.8 mm (range 0.8–4.4 mm; 95% CI: [1.7 mm; 2.3 mm]) for the ovoids screwed to the intrauterine probes, as well as of 1.8 ± 0.9 mm (range 0.8–3.6 mm; 95% CI: [1.4 mm; 2.2 mm]) for the needles. Mean deviations with MAE ≤1, ≤2, and ≤25 mm were observed in 6%, 83%, and 89% of all cases, respectively. The reported outliers could be traced back to variabilities in reconstructions or a larger distance of needle courses to the probe (associated with stronger deviations caused by even slight rotational image registration errors). The results thus represented a reasonable agreement between the applicator courses of intraoperative and postoperative imaging.

## 4 Discussion

In the frame of the present work, we implemented intraoperative CBCT as an additional part of our institution’s default workflow for the brachytherapy of cervical cancer. The establishment of the individual workflow steps was described in detail, accompanied by our single-center experience report on the respective (dis)advantages. Our aim was thereby to provide a corresponding recommendation for introducing intraoperative CBCT in other institutions as well. In particular, we also conducted a quantitative assessment of image quality as well as of the geometric and dosimetric benefits associated with using this modality in the described setting. Although some hospitals already use CBCT or further cross-sectional imaging, e.g., MRI during surgery (17, 18), there exist to our knowledge currently no detailed descriptions of the establishment of such workflows in combination with a comprehensive assessment of the associated benefits and drawbacks. In particular, the ImagingRing has so far only been characterized in preclinical studies (25–27) and applied for treatment QA (16, 35), but its utilization for intraoperative purposes has not been reported so far.

In terms of image quality, a clear delineation of tissue structures and reconstructability of implanted applicators was obtained for 20 of the 26 patients receiving CBCT. This was confirmed by the significant applicator differentiability from the image background and the reasonable CNR of 5.1 ± 3.2 between the bladder and uterus as well as 8.5 ± 3.6 between the uterus and visceral fat. In this regard, the measurements fulfilled Rose’s criterion (36) stating that structures are in general distinguishable from each other if their CNR exceeds a value of 3 to 5. Nevertheless, it has to be mentioned that the CNR of CBCT scans is in general lower compared to that of conventional CT (37, 38) due to the increased X-ray scatter. Our investigations could not confirm the bad image quality with median CNRs of only 2.1 (range 0.2–4.8) and extensively occurring artifacts reported for previous ImagingRing applications for treatment QA (16), meaning that substantial improvements of the respective imaging performance (27) had to be carried out in the meantime. In the present work, artifacts were limited but particularly pronounced when metal needles were used. Consequently, the combined use of metal needles with the ImagingRing is not recommended. Apart from this drawback, the obtained image quality was deemed sufficient for the purpose of intraoperative image guidance in general. The associated dose exposure (mean CBDI_w_ of 9.2 ± 1.3 mGy per scan) was comparable to that of conventional CT (39, 40) and very small compared to the prescribed treatment doses of up to 45 Gy but remains an important factor to be considered in clinical practice. However, it has to be mentioned that intraoperative imaging by means of dose-neutral MRI would be strongly preferable due to the substantially enhanced soft-tissue contrast (11, 41, 42) achievable with this modality but for logistical reasons (e.g., requirement of an MR-safe surgical theatre, no mobile devices available, and increased time requirements) is not implementable at most centers worldwide. In these cases, mobile CBCT can support the creation of a high implant quality as shown in this work.

The implementation of CBCT into our workflow resulted in additional needle shifts with a mean EID of 14 ± 11 mm into the pelvis and a reduction of needles featuring a too-low insertion depth from 18% to only 5%. This led to statistically significant dosimetric improvements considering both the cohort-based and patient-individual analyses. Note that the actual clinical relevance of these improvements regarding clinical outcomes can only be evaluated in future studies comparing tumor control and toxicity profiles, which was beyond the scope of the present work. Although our evaluation, as well as the clinical experience and several descriptions in literature (12, 43, 44), showed that achieving a very good implant and treatment is possible by means of sole ultrasound guidance in principle, these could be further optimized considering intraoperative CBCT. While the experience and ability of a brachytherapist are considered most relevant to creating a suitable implant, any additional support in implantation guidance is deemed important to ensure the safety of the procedure and to be able to adapt the implant to patient-specific clinical requirements. For this reason, intraoperative CBCT has gained high value in our institution since its introduction. In particular, our professional experience in gynecologic brachytherapy reaches up to 30 years in our team, and younger or less experienced physicians may profit even more by considering intraoperative CBCT images in general. However, with respect to the performed dosimetric assessments, it has to be mentioned that dose distribution planning and the final assessment of the treatment plan are processes affected by observer variability (which holds for both manual forward planning and inverse planning techniques (45–47)), and the relation between improved geometric implant arrangement and dose distribution is generally complex. For instance, we showed that using virtually shifted, suboptimal-positioned needles to achieve a comparable dose coverage of the target volume was still feasible by expanding the dwell times of individual dwell positions of the afterloader source. However, this achievement was associated with increased exposure to OARs, which is why the resulting tradeoff between OAR and target volume has to be clinically considered for each patient. In the present analysis, we showed that this tradeoff could be improved by considering additional intraoperative CBCT. In particular, our approach of performing both a dose comparison of cohorts and patient-individual recalculations exploited the possibilities for a comprehensive assessment of the dosimetric benefits associated with the workflow change. The provided results refer to single-center experiences and have to be validated in further studies by institutions to derive a potential general validity of our statements. Nevertheless, our investigations indicated the advantages of utilizing cross-sectional 3D imaging in the intraoperative setting and can support other sites in their endeavors in this regard.

The establishment of the new workflow was associated with the challenges reported above, such as a restriction of the working space of surgical nurses, the requirements for patient position changes for imaging, and an increased time effort, particularly in case of multiple repetitive CBCT scans being required. Focusing on the best possible adaption of the implantation to the underlying patient, these challenges and efforts were definitely justifiable and in the interest of the patient. Appropriate organizational changes to integrate the increased intervention time into the clinical routine should be pursued on an institute-specific level. However, to address the disadvantages of required patient position changes and repetitive imaging, CBCT-related optical tracking as an additional solution for guiding implantations of rigid applicators forms a subject of ongoing research (22, 48). A corresponding approach aims to project needle courses *in situ* into the anatomical information gained from one single CBCT scan acquired at the beginning of the interventional procedure but is still under pre-clinical investigation (48).

With a mean MAE of 2.0 ± 0.8 mm for the ovoids rigidly screwed to the intrauterine probes and 1.8 ± 0.9 mm for interstitial needles, the CBCT scans showed a reasonable imaging fidelity of the implant arrangement with respect to the planning imaging. Considering an inter-observer variability in applicator reconstruction on both CBCTs and planning CTs of approximately 0.60 ± 0.35 mm (49), the low resolution of the planning MRIs with voxel sizes of 1 × 1 × 1 mm^3^ associated with partial volume effects, and image registration uncertainties with a MAE of 0.41 ± 0.12 mm obtained for the intrauterine probes, the relative spatial deviations reported for ovoids and needles were deemed reasonable. In particular, they were smaller than one afterloader step size of 2.5 mm. The impact of patient position changes, patient transfer, or potential geometric image distortions on the reconstructed implant arrangement was therefore considered limited. It has to be noted that the performed analysis only compared applicator courses and did not address implant deviations in relation to anatomy, but a slippage of the entire applicator arrangement in one direction was considered clinically unlikely due to the placed very firm tamponade and was also not observed in the assessments of the intraoperative and planning scans. Based on the reported reasonable imaging fidelity, we consider the potential use of the intraoperative CBCT scans for the purpose of treatment planning feasible. Although currently not implemented in our routine, this could avoid the acquisition of additional planning imaging after surgery and even reduce the total time required from anesthesia induction to treatment delivery despite the additional intraoperative workflow steps in some cases. However, respective investigations have to form the subject of future studies.

In summary, the implementation of intraoperative CBCT into our workflow for the brachytherapy of cervical cancer resulted in geometric and dosimetric improvements in the individual brachytherapy procedure and as a consequence is beneficial for the patients. Further research beyond the scope of our single-center experience is encouraged to confirm this assessment. In particular, while the number of 26 patients examined in this work is considered reasonable to report a primary experience and perform a first benefit assessment, larger studies could enhance the robustness and generalizability of the findings and are therefore aimed at. Furthermore, it will be intriguing to investigate in the future whether these dosimetric advantages translate into improved clinical outcomes and reduced treatment-related side effects. Nevertheless, although associated with additional effort and time requirements, the introduction of corresponding workflows allows for increased precision, safety, and efficacy of interventional brachytherapy procedures. The present manuscript can in particular serve as a recommendation for the implementation of similar workflows in further institutions.

## Data Availability

The raw data supporting the conclusions of this article will be made available by the authors upon reasonable request.
